# Electrolyte Disturbances in SARS-CoV-2 Infection

**DOI:** 10.12688/f1000research.24441.2

**Published:** 2020-07-22

**Authors:** Holly Mabillard, John A. Sayer

**Affiliations:** 1 Renal Services, The Newcastle Hospitals NHS Foundation Trust, Newacstle upon Tyne, Tyne and Wear, NE77DN, UK; 2 Translational and Clinical Research Institute, Newcastle University, Newcastle upon Tyne, Tyne and Wear, NE13BZ, UK; 3 NIHR Newcastle Biomedical Research Centre, Newcastle University, Newcastle upon Tyne, Tyne and Wear, NE45PL, UK

**Keywords:** Hypokalaemia, SARS-CoV-2, ACE-2, transtubular potassium gradient, alkalosis

## Abstract

The global pandemic secondary to the severe acute respiratory syndrome coronavirus 2 (SARS-CoV-2) is leading to unprecedented global morbidity and mortality. With a bewildering array of complications, renal involvement in various forms is common, including serum electrolyte derangements. Hypokalaemia secondary to SARS-CoV-2 was common in a reported Chinese cohort. Here we review the emerging evidence on hypokalaemia and SARS-CoV-2 infection, the potential pathophysiological mechanisms based on early clinical and histopathological data and important clinical implications. Mechanisms of hypokalaemia are multifactorial and so the electrolyte disturbance can be difficult to avoid. We provide further support to the theory of renin-angiotensin-aldosterone (RAS) activation, discuss the strengths and weaknesses of implicating RAS involvement and highlight the importance of calculating the transtubular potassium gradient to identify those at risk of hypokalaemia and its complications.

## Introduction

The global pandemic secondary to the severe acute respiratory syndrome coronavirus 2 (SARS-CoV-2) is leading to unprecedented global morbidity and mortality. Common symptoms include fever (43% of patients), cough (50%) and dyspnoea (29%) but other features such as myalgia (36%), diarrhoea (19%), anosmia and hypogeusia (10%) are also common ^1^. The most frequent serious manifestation of infection is pneumonia with 15% of patients developing serious manifestations such as hypoxia, dyspnoea, extensive pulmonary involvement and acute respiratory distress syndrome (ARDS) ^2– 5^. A recent meta-analysis including 50,466 patients with SARS-CoV-2 infection described an ARDS incidence of 14.8% ^6^. Anecdotal data and clinical observation from patients with the World Health Organization named disease coronavirus disease 2019 (COVID-19) has highlighted a bewildering array of presentations and pathologies in those infected with SAR-CoV-2 many of which, it should be stressed, are rare. These include additional respiratory complications (pulmonary fibrosis – reported in 21% of those hospitalised with SARS-CoV-2 9 months post-discharge in one study ^3^) ^7^, cardiovascular complications (acute cardiac injury (7% ^8^), cardiomyopathy (1/3 patients ^9^), cardiac tamponade, heart failure, dysrhythmias (17% ^8^) and venous thromboembolic events (20% ^10^)) ^11^, neurological complications (myopathy, acute stroke (5.7% of those with severe infection ^12^), Guillain-Barre syndrome (0.4% hospitalised patients ^11^) and encephalopathy) ^13^, acute liver and/or pancreatic injury (29% and 17% respectively in one cohort) ^14^, cytokine storm syndrome, septic shock, DIC, diarrhoea, Kawasaki-like disease ^14^ and renal complications (acute tubular injury, rhabdomyolysis, proteinuria, secondary focal segmental glomerulosclerosis and possible renin-angiotensin-aldosterone system activation) ^15^.

A few small studies have demonstrated that SARS-CoV-2 can affect the kidneys directly but it is unclear if this is a key mechanism in acute kidney injury seen in patients with SARS-CoV-2 infection. Involvement of the renin-angiotensin-aldosterone (RAS) pathway has been speculated as a mechanism to lead to more severe COVID-19 disease and patients with severe disease were more likely to have hypertension, chronic kidney disease (CKD), diabetes mellitus and cardiovascular disease than those with milder disease. This is, however not confirmed and is preliminary data that may be the subject of bias with disregard of age as a potential confounding factor ^16^. Here we aim to review the evidence, potential clinical implications and possible mechanisms of electrolyte disturbances and kidney injury in patients with SARS-CoV-2 infection.

Firstly, it is important to highlight how transmission of the virus occurs in humans. The SARS-CoV-2 spike protein enters host cells via the angiotensin-converting enzyme 2 (ACE2) receptor on the surface of pulmonary type 2 alveolar cells ^16, 17^. ACE2 is crucial to counter-regulate RAS and shares approximately 60% homology with ACE. RAS pathway components are co-expressed in most organs and tissues in the body and communicate via both paracrine and autocrine signalling. ACE2 converts angiotensin-II into angiotensin-(1-7), which acts on the Mas receptor expressed on a variety of tissue cell lineages relevant to cardiovascular disease in addition to type 2 alveolar epithelial cells. It lowers blood pressure through vasodilation and promotion of renal sodium and water excretion and it also attenuates inflammation by producing nitric oxide ^18^. Meanwhile, ACE converts angiotensin-I to angiotensin-II which has directly opposing effects to signalling mediated by ACE2. Angiotensin-II acts at the type 1 angiotensin receptor (AT1R) to increase blood pressure by the induction of vasoconstriction and renal sodium and water reabsorption. This also creates oxidative stress which promotes inflammation and fibrosis. The balance between these two opposing pathways determines potential tissue injury, predominantly in the heart and kidneys ^16, 17^. Not only has the ACE2 receptor found to be used for host cell entry by the SARS-CoV virus, but the affinity for SARS-CoV-2 is 10-20-fold higher ^18^. During host cell entry, proteolytic cleavage of ACE2 by transmembrane serine protease 2 (TMPRSS2) occurs which is thought to suppress ACE2 expression. This in theory would lead to a reduction in angiotensin-(1-7) generation and angiotensin-II levels would increase resulting in oxidative-stress mediated tissue damage and hypertension ^16, 17^. This mechanism resulting in lung parenchymal injury has already been demonstrated in mice injected with SARS-CoV virus ^19^.

Involvement of the RAS pathway has therefore been speculated as a mechanism to lead to more severe COVID-19 disease and patients with severe disease were more likely to have hypertension, chronic kidney disease (CKD), diabetes mellitus and cardiovascular disease than those with milder disease. This is, however not confirmed and is preliminary data that may be the subject of bias with disregard of age as a potential confounding factor ^17^.

Hypokalaemia is known to cause muscle weakness, paraesthesia, thirst, muscle cramps and weakness, but hypokalaemia is an important complication of any disease due to it being a potentially life-threatening condition ^20^. In fact, the incidence of ventricular fibrillation has been shown to be fivefold higher in patients with hypokalaemia compared to those with hyperkalaemia ^21^. Furthermore, inadequate management of hypokalaemia has been identified in 24% of hospitalised patients ^22^, reiterating that we need to be even more vigilant with the management of this life-threatening condition during a pandemic where resources are even more scarce.

### What is the evidence for hypokalaemia in SARS-CoV-2 infection so far?

The release of a pre-print retrospective Chinese study initially sparked interest into hypokalaemia as a potentially prevalent biochemical disturbance in SARS-CoV-2 infection ^23^. This was released around a similar time to concerns around RAS inhibitors and SARS-CoV-2 where speculation that RAS inhibitors may increase risk of SARS-CoV-2 infection and its severity due to the knowledge that ACE2 is the viral binding site for host cell entry. It has been stressed by multiple organisations globally that RAS inhibitors should be continued unless otherwise medially indicated as the risks of stopping these important drugs have numerous serious complications to the patient. This study claimed that hypokalaemia was prevalent amongst SARS-CoV-2 infected patients, affecting 108 of 175 patients (62%) that had a serum potassium of <3.5 mmol/l and only 10 patients had a serum K >4.0 mmol/l which is the value required for those with myocardial dysfunction. Of the patients, 22% had severe hypokalaemia (serum potassium <3.0 mmol/l). In total, 11% of all patients and 28% of those with severe hypokalaemia displayed metabolic alkalosis (pH >7.45) compared to 4% with normokalaemia suggesting the possibility of RAS activation. The study suggested that the hypokalaemia was not due to gastrointestinal (GI) loss of potassium as only a small proportion of patients had GI symptoms, there was no significant difference between serum potassium in those with or without diarrhoea and significant urinary potassium wasting was shown. A total of 20 patients had a spot urine potassium/creatinine ratio and there were significantly higher urinary potassium/creatinine ratios in those with hypokalaemia compared with normokalaemia. The study reported that the degree of hypokalaemia correlated with severity of SARS-CoV-2 symptoms and they suggested that hypokalaemia can be difficult to correct as seen in two patients because the renal potassium wasting persists until clinical recovery from the virus. This study is relatively small, limited to a Chinese population and did not disclose medications or diuretic use which could be a significant confounding factor here. As diuretics (especially “loop” diuretics) are used to improve oxygenation in patients with ARDS, some of these patients may well have been prescribed them. It was postulated that hypokalaemia in these patients is due to the effects of increased angiotensin-2 resulting from the proteolytic cleavage of ACE2 from virion invasion of the host although this conclusion cannot be made without measuring components of the RAS system in these patients. Further studies including declaration of medications/diuretic use and measurement of components of the RAS pathway are necessary to make conclusions about aetiology of hypokalaemia in SARS-CoV-2 infection although hypokalaemia was clearly a clinical risk in these patients that should be communicated to the medical community.

A study performed in Hong Kong during the 2003 SARS-CoV epidemic compared symptoms and laboratory findings in SARS-CoV RT-PCR swab-positive patients to negative patients ^24^. Hypokalaemia was present in 41.3% of RT-PCR positive patients and was reported as a ‘common laboratory finding’. It was also suggested in this paper that the hypokalaemia was due to the increased effects of angiotensin-2 but, again, the RAS components were not measured in patients. In contrast, the largest SARS-CoV-2 case series so far (1099 patients included) did not demonstrate any major difference in serum potassium between those with mild and those with severe disease ^2^. Serum potassium was mostly reported as normal in this cohort. SARS-CoV was found in the urine in one patient but urine was not routinely tested for the virus in this cohort.

A small Chinese, retrospective cohort of 12 patients who were hospitalised with varying degrees of severity of SARS-CoV-2 infection were studied to comprehensively assess renal function, risk factors for, and incidence of early renal injury. 50% of patients had hypokalaemia and 50% also had hyponatraemia ^25^. However, this study did not acknowledge if any of the patients were taking diuretics or other medications that could alter serum potassium or sodium concentration.

### Not all patients with hyperaldosteronism develop hypokalaemia

**Figure 1.**
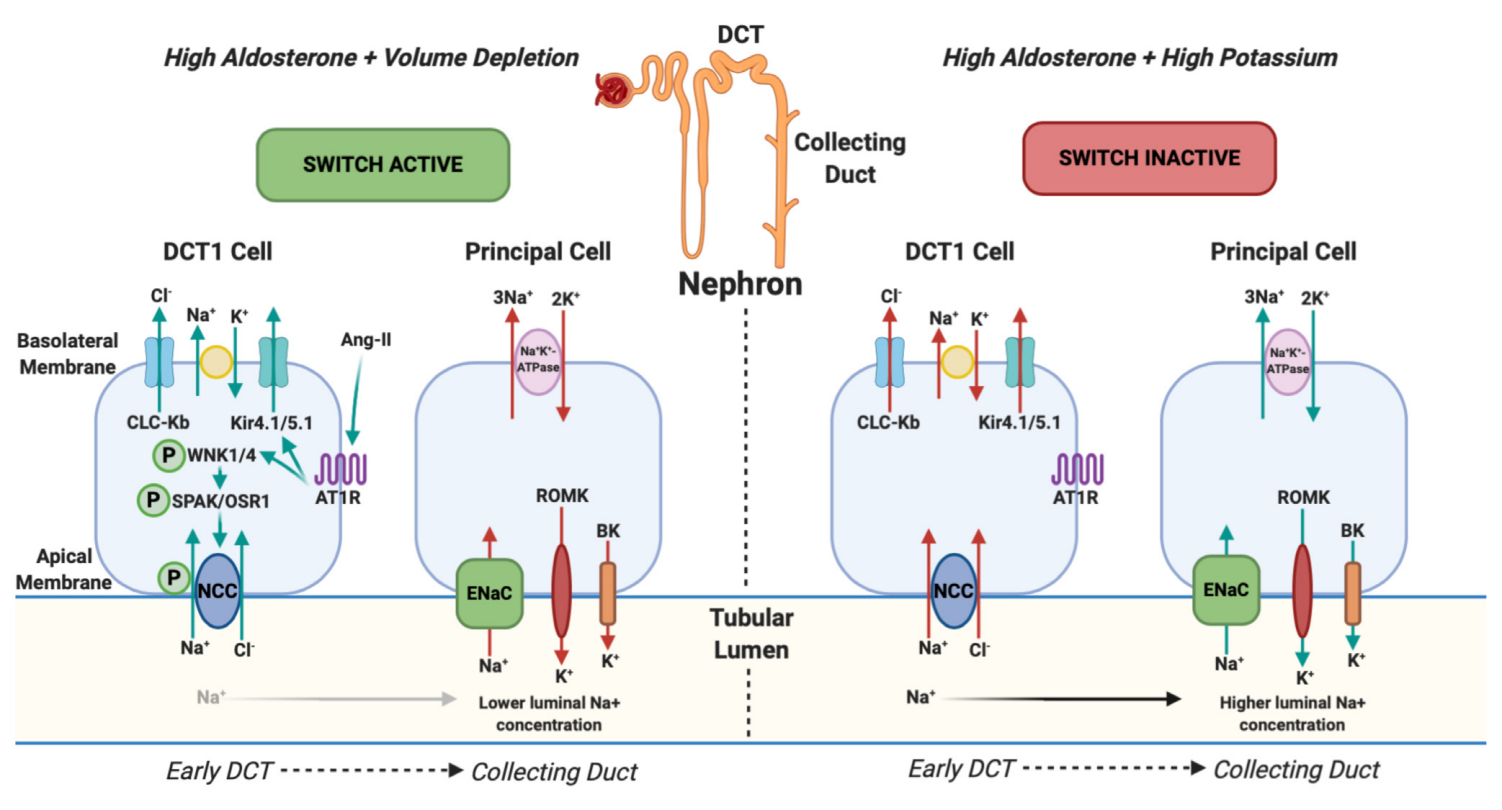
NCC Phosphorylation downstream and changes in urinary potassium (renal potassium switch). In states of hyperaldosteronism and volume depletion the renal potassium switch is ‘active’. Angiotensin-II (ang-II) stimulates Kir4.1/5.1 to hyperpolarise the basolateral membrane potential and lowers intracellular chloride. This action activates WNK1 (With-no-lysine-kinase-1) and WNK4 (With-no-lysine-kinase-4) by phosphorylation which subsequently phosphorylates SPS1-related proline / alanine-rich kinase (SPAK) and oxidative stress-responsive kinase (OSR1) which, again, phosphorylates the sodium chloride co-symporter (NCC). NCC then avidly reabsorbs sodium (and chloride) back into the body in the early part of the distal convoluted tubule (DCT1). This results in reduced tubular sodium delivery to the collecting duct. Subsequently there is less sodium for the Epithelial sodium Channel (ENaC) to reabsorb and its action is offset. ENaC usually reabsorbs sodium and, via an electrochemical gradient made by sodium-potassium-ATPase, potassium is normally excreted into the tubular lumen by ROMK (Renal Outer Medulla Potassium channel) and BK (Big Potassium/Maxi-K channel) and eliminated from the body. When ENaC is less active, as a consequence of less tubular sodium delivery, less potassium is excreted. This results in a state of hyperaldosteronism with a normal serum potassium. In states of hyperaldosteronism and normal cardiovascular volume (in response to high serum potassium) the switch is ‘inactive’. NCC is not phosphorylated which results in more distal sodium delivery to the collecting duct and subsequent ENaC activation resulting in excretion of potassium into the tubular lumen and a state of hypokalaemia ^26, 27^. Arrows represent the direction of movement of electrolytes. Red arrows reflect a reduction in movement and green arrows represent an increase in movement. CLC-Kb, chloride voltage-gated channel Kb; Kir4.1/5.1, inwardly rectifying potassium channel 4.1; AT1R, type 1 angiotensin receptor.

The theory of RAS activation causing hypokalaemia is popular, however it is recognised that hyperaldosteronism does not always cause hypokalaemia. This is explained by the ‘renal potassium switch’ mechanism ( Figure 1), which involves the paradoxical actions of aldosterone on potassium; for example, aldosterone increases sodium reabsorption in states of low circulating volume without significantly altering potassium balance, but it also increases serum potassium excretion in high potassium states without affecting sodium balance. This paradoxical action all depends on the sodium delivery to the aldosterone-sensitive distal nephron (collecting duct) which is generated by the sodium-chloride co-symporter (NCC) in the early distal convoluted tubule. Activation of this ‘switch’ is by phosphorylation of NCC by the WNK/SPAK/OSR1 pathway ^26, 27^ and is done by aldosterone in response to low or high serum potassium i.e. NCC is maximally phosphorylated when serum potassium is <2.5 mmol/l and not at all phosphorylated when serum potassium is >5.2 mmol/l ^28^. In states of decreased circulating volume, ENaC activity is increased due to the effects of excess aldosterone (which generates a sufficient intracellular electrochemical gradient to cause active transport of potassium from blood via Na ^+^K ^+^-ATPase which would be passively excreted into the tubular lumen by ROMK and BK channels in the Principal cell). Whether this generates an effect on potassium or not depends on the integrated activity of the entire distal nephron. In a state of low circulating volume, NCC in the early DCT is ‘switched on’ which results in increased reabsorption of sodium and chloride in the early DCT and, along with sodium reabsorption in the PCT, a net increase of sodium reabsorption from the kidney occurs. As a result, sodium delivery to the aldosterone sensitive distal nephron (collecting duct) is decreased which offsets the effects of ENaC and potassium excretion is therefore unchanged. This results in a state of hyperaldosteronism without hypokalaemia ^28^. It should be noted that, although the renal potassium ‘switch’ mechanism is a popular theory, it has never been proven although there are case reports of patients with normokalaemic primary hyperaldosteronism that develop hypokalaemia with a thiazide diuretic ^29^.

### Other electrolyte disturbances

Various case reports describe the onset of Syndrome of inappropriate anti-diuretic hormone secretion (SiADH) in some patients with SARS-CoV-2 infection ^30^. Although SiADH is frequently associated with atypical pneumonia and pneumonia is a frequent complication of SARS-CoV-2 infection, the literature does describe this phenomenon in the absence of respiratory symptoms, fever and any alternative explanation for SiADH ^31^. It is therefore prudent to consider testing for SARS-CoV-2 infection in cases of SiADH without a clear aetiology. Additional studies are required to ascertain incidence and pathogenesis of SiADH in SARS-CoV-2 infection.

One retrospective cohort study of 42 laboratory-confirmed COVID-19 patients without a history of chronic kidney disease have shown a high incidence of novel acquired incomplete renal Fanconi syndrome, often preceding acute kidney injury and/or resolving during the clinical recovery phase of the infection ^32^. 75% of patients admitted to hospital for mild, moderate or severe respiratory failure due to SARS-CoV-2 infection had acute incomplete renal Fanconi syndrome. Proteinuria was always associated and often with severe renal phosphate leak (in addition to frequent hypophosphataemia, hyperuricosuria and glycosuria). Renal associated hypokalaemia and renal tubular acidosis was not analysed in this study due to many patients requiring potassium supplementation in addition to the effects of mechanical ventilation, GFR alterations and other factors. The authors suggest that the development of incomplete renal Fanconi syndrome could be used as a predictor of acute kidney injury (and a prognostic marker) as 88% of patients with severe stage 2 and 3 KDIGO acute kidney injury experienced proximal tubule injury prior to acute kidney injury onset. None of these patients had significant haemodynamic instability or had high mean SOFA scores to suggest an alternative pre-renal aetiology for their acute kidney injury. A quarter of the patients were given Lopinavir/ritonavir but this is rarely associated with acute kidney injury and not associated with renal Fanconi syndrome.

### Histopathological evidence of tubulopathy

There are two studies so far (one in pre-print form) to look at renal histopathology on post-mortem findings in SARS-CoV-2 patients who died of the disease (9 of the 26 had pre-morbid clinical kidney injury signs) ^33, 34^. The first demonstrated prominent proximal tubule injury (observed in all 26 patients) with frank necrosis observed in some specimens and three patients had high creatinine phosphokinase and pigmented casts on light microscopy, probably representing rhabdomyolysis. There was occasional distal tubule and collecting duct swelling with some interstitial oedema and virus particles were seen in seven of the specimens on electron microscopy in proximal tubule epithelial cells and the podocyte, both of which are the only sites of renal ACE2 expression ^33^. The second post-mortem case series in (currently in pre-print) examined six post-mortem renal histopathology samples of patients who died with SARS-CoV-2 infection ^34^. All specimens had histopathological evidence of acute tubular necrosis, two patients had severe lymphocytic infiltration of the tubulointerstitium and SARS-CoV-2 nucleocapsid antigens were observed in renal tubule cells. Transmission electron microscopy was used in samples from two different patients, both of which demonstrated the presence of virions and virus-like particles in renal cells and these cells were markedly swollen with mitochondrial, lysosomal and endoplasmic reticulum expansion. These finding suggest that SARS-CoV-2 directly infects kidneys and specifically infects and damages kidney tubules. Furthermore, strong CD68 ^+^ macrophage presence and C5b-9 deposition seen in the tubulointerstitium of all samples (yet absent in the rest of the kidney tissue and very little in the glomeruli and capillaries in two patients) suggest that further tubular damage occurs as a consequence of macrophage recruitment and C5b-9 activation and deposition. It would be logical to suggest that histopathological evidence of damage to tubular cells would result, clinically, as a tubulopathy, of which we know urine electrolyte wasting is a recognised complication and these case series are histopathological evidence to support this suggestion.

Finally, we know that TMPRSS2 primes SARS-CoV-2 to gain cellular entry and TMPRSS2 is only detectable (in low levels) at the S3 section of the proximal convoluted tubule in the kidney ^35^. It is therefore assumed that this is one way in which the kidney is ‘infected’ by the virus and reiterates the probability of direct virus-induced tubular damage. Various case series have demonstrated the virus in urine ^36, 37^ and it is unclear as to how it enters the tubular lumen and whether this is via the apical membrane of tubule cells. However, one study did look at the presence of SARS-CoV-2 in bodily fluids over time and did not detect the virus in urine in any of the nine patients studied ^38^. A couple of case reports have demonstrated that the virus may affect the kidney in other ways which may be an alternative explanation for the entry of virus into the urine ^39, 40^. Collapsing focal segmental glomerulosclerosis (FSGS) has been described twice in the literature so far in patients of African or African American origin and both of these patients demonstrated severe tubular injury. It was noted that severe acute tubular necrosis (ATN) was observed in the absence of haemodynamic compromise and severe pulmonary involvement suggesting that the tubulopathy was not ischaemic and more likely to be because of either direct viral toxicity or cytokine-mediated damage.

## Discussion

Various clinical and histopathological studies have demonstrated evidence of hypokalaemia, hyponatraemia, SiADH, incomplete Fanconi syndrome and tubulopathy in patients with SARS-CoV-2 infection.

The data from China and evidence from the SARS-CoV case series provide good clinical justification to monitor potassium levels in patients infected with SARS-CoV-2. We do need to see this clinical finding replicated in multi-centres to be able to make definitive conclusions about this clinical sequela. Although hyperkalaemia can occur for many reasons in patients with SARS-CoV-2 infection, the incidence of ventricular fibrillation has been shown to be five-fold higher in patients with hypokalaemia compared to those with hyperkalaemia ^21^.

Based on evidence of significant urine potassium wasting which can be prolonged, we suggest checking the transtubular potassium gradient (TTKG) in patients with SARS-CoV-2 infection. The TTKG adjusts the urinary potassium for the concentrating effects that occur in the collecting duct where water is removed from urine. This would help identify those at risk of severe or prolonged hypokalaemia and prompt pre-emptive cautious electrolyte monitoring and replacement which should help reduce the risk of hypokalaemic complications which can be fatal. The TTKG is easily measured and only requires measurements of serum and urine osmolality and potassium (urine potassium/serum potassium)/(urine osmolality/ serum osmolality). A high TTKG in the setting of hypokalaemia suggests that potassium wasting is of renal origin. This, for example, may be due to hyperaldosteronism, pseudohyperaldosteronism or a potassium wasting tubulopathy. The validity of this measurement depends on three assumptions: (1) few solutes are reabsorbed in the medullary collecting duct, (2) potassium is neither secreted or reabsorbed in the medullary collecting duct and (3) the osmolality of fluid in the terminal collecting duct is iso-osmolar to plasma. As water is reabsorbed in the medullary collecting duct, urine potassium concentration is not an accurate index evaluating distal potassium secretion because the effect of water is not taken into account. The urine to plasma osmolality ratio adjusts for the degree of medullary water reabsorption which increases urine potassium concentration as more water is reabsorbed. Therefore, TTKG is relatively accurate providing the urine is not dilute, that the osmolality of urine is greater or equal to the osmolality of plasma (because vasopressin is required for optimal potassium excretion in the distal nephron) and that urine sodium concentration is >25mmol/l so that distal sodium delivery is not limiting ^41^. The main premise underlying the TTKG is the absence of significant solute transport in the collecting duct so that any change in urinary potassium concentration only occurs due to medullary water reabsorption and the TTKG does not overestimate the gradient for collecting duct potassium secretion. However, we now know that some urea is reabsorbed in the late cortical collecting duct and this urea recycling aids the tubular secretion of potassium so it should be noted that the TTKG should not be used as a diagnostic tool in hyperkalaemia aetiology as it is not reliable ^42^. In situations of dilute urine and high urine flow rate, the TTKG also underestimates potassium secretory capacity in the hyperkalaemic patient ^43^. However, we believe that the utility of the TTKG is still of value in hypokalaemic patients to differentiate between renal and extra-renal potassium wasting providing the aforementioned criteria necessitating its accuracy is adhered to.

Measuring components of the renin-angiotensin-aldosterone pathway would help us to identify if there really is involvement of the SARS-CoV-2 virus; however, measuring these components in the acute setting has its limitations. The RAS system is heavily influenced by a multitude of factors. Many of the patients with SARS-CoV-2 infection are critically unwell and often present to hospital in states of low cardiovascular volume. It has been recommended that patients with ARDS also be kept in a relative state of volume depletion as to not worsen pulmonary interstitial oedema and thus oxygenation. Both of these factors would typically result in RAS activation and could be very misleading when determining virus-related RAS pathway involvement. Some studies have shown that low urine pH (causing acidic urine) can be a predictor of RAS activation ^44^ so future studies determining RAS system involvement of SARS-CoV-2 infection should, as well as TTKG, also consider urine pH as an early marker of RAS system activation.

Potassium levels <3.0 mmol/l can be arrhythmogenic and specifically can cause QTc interval prolongation, torsade de pointes, ventricular fibrillation and sudden cardiac death ^45^. This is particularly relevant for a couple of reasons. First, many of the drugs currently undergoing clinical trials in SARS-CoV-2 patients prolong the QTc interval (hydroxychloroquine ^46^, azithromycin ^46^, favipiravir, lopinavir/ritonavir and fingolimod: NCT04280588) and some drugs also risk hypokalaemia which may worsen pre-existing electrolyte disturbance (e.g. methylprednisolone: NCT04273321, NCT04263402; thalidomide: NCT04273529, NCT04273581; and bevacizumab: NCT04275414). Second, cardiac involvement in SARS-CoV-2 is high (44.4% of infected patients admitted to ICU experienced an arrhythmia). Induction of arrhythmias can be due to multi-factorial aetiologies of cardiac injury in SARS-CoV-2 patients such as hypoxia-mediated, direct tissue damage, cytokine-storm syndrome, worsening coronary perfusion and the direct effects of medications ^47^. Both of these factors really emphasise the importance of maintaining normokalaemia in these patients to reduce morbidity and mortality.

In addition to the arrhythmogenic effects of both the SARS-CoV-2 cardiac sequelae and various clinical trial drugs, many patients are being given diuretics to improve the oxygenation in ARDS, which also risks hypokalaemic complications. Loop diuretics can induce hypokalaemia by blocking the Na ^+^-K ^+^-2Cl ^-^ co-transporter (NKCC2) in the thick ascending limb of the loop of Henle which results in failure of sodium, potassium and chloride to be reabsorbed into the concentrated medullary interstitium (this transporter normally reabsorbs about 25% of the sodium load). This enhances distal tubular concentration of sodium, reduced hypertonicity of the surrounding interstitium and less water reabsorption in the collecting duct. Distal sodium delivery increases potassium loss via the Na+/K+-ATPase pumps at the apical membrane of the principal cell of the collecting duct as there is more sodium to be exchanged with potassium for excretion. Thiazide diuretics can cause hypokalaemia by the same principle of enhanced distal sodium delivery as they block the sodium-chloride co-symporter (NCC) in the distal convoluted tubule. Furthermore, loop diuretics also block NKCC2 at the macula densa which (along with the RAS-activation response to initial volume reduction) induces renin secretion, making RAS system measurement inaccurate if patients are on diuretics. If diuretics are to be used, it would be wise to consider potassium-sparing agents in hypokalaemic patients to reduce the cardiac complications of worsening hypokalaemia that can occur with those that are not potassium sparing.

It would be logical that with clear histopathological data suggesting tubular injury, hypokalaemia is to be expected in patients infected with SARS-CoV-2 ^48^. Tubular injury seems to be multifactorial in these patients; ATN from SIRS/Cytokine Storm Syndrome, peritubular congestion, virion invasion, drug toxicity and pigment-cast nephropathy from rhabdomyolysis and therefore difficult to avoid.

The data on outcomes in cardiac arrest for SARS-CoV-2 patients is unfortunately poor. Chinese data suggests that in a series of 136 patients who underwent resuscitation efforts, return of spontaneous circulation was only achieved in 18 (13.2%) and only four patients were alive at 30 days ^49^. The strong association of hypokalaemia with arrhythmia and sudden cardiac death reiterates the importance of vigilance for detection and treatment of this electrolyte disturbance during and after admission given such poor outcomes when spontaneous circulation is lost.

To truly know the extent of involvement of the RAS pathway as a potential cause of hypokalaemia in SARS-CoV-2 patients, further studies require measurement of all components of the RAS pathway. Until we know this, we cannot make definite conclusions about the mechanisms of hypokalaemia in these patients outside of the histopathological evidence of tubulopathy published, of which multiple aetiological factors are likely.

## Data availability

No data are associated with this article.
