# Supplementary material for: Electrolyte Disturbances in SARS-CoV-2 Infection
Source: F1000Res. 2020 Jul 22;9:587. Originally published 2020 Jun 10. [Version 2] doi: 10.12688/f1000research.24441.2 (PMC7533733; doi:10.12688/f1000research.24441.2)
Supplement: Peer review history

# Peer review: Reviewer response for version 2

**DOI:** 10.5256/f1000research.27922.r70112

**Published:** 2020-10-02

**Authors:** Stephen B. Walsh, Elizabeth Wan

**Affiliations:** 1 University College London , London, UK

**Summary**

 This is a narrative review of hypokalaemia experienced in SARS-CoV-2 infection.

**Strengths**

 The paper addresses available literature.

 The paper correctly critiques the level of evidence included, which for the most part includes small and poorly conducted observational studies.

**Major weaknesses**

 None

**Minor Weaknesses**

 Some aspects of the writing were difficult to follow

- Please broaden abstract to include electrolyte disturbances in addition to hypokalaemia, reflecting the article as a whole.
- In the first paragraph, there is quite a lot of text dealing with the many complications of COVID infection, which is a bit irrelevant.
- “…proximal tubule epithelial cells and the podocyte, both of which are the only sites of renal ACE2 expression” This is not true; see The Human Protein Atlas or for example ^1^.
- “A few small studies have demonstrated that SARS-CoV-2 can affect the kidneys directly, but it is unclear if this is a key mechanism in acute kidney injury seen in patients with SARS-CoV-2 infection”? Reference. It would be useful to include a comment on the prevalence of acute kidney injury in SARS-CoV-2 patients.
- “Hypokalaemia is known to cause muscle weakness, paraesthesia, thirst, muscle cramps and weakness, but hypokalaemia is an important complication of any disease due to it being a potentially life-threatening condition”. Please rephrase – hypokalaemia is important, but at the same time rare (does not occur with ‘any disease’).
- “Furthermore, inadequate management of hypokalaemia has been identified in 24% of hospitalised patients ^22^, reiterating that we need to be even more vigilant with the management of this life-threatening condition during a pandemic where resources are even more scarce”. Please reference, or clarify. Management of hypokalaemia is not usually resource-intensive, and I have not seen evidence that potassium replacement is difficult to come by in the pandemic.
- “This study claimed that hypokalaemia was prevalent amongst SARS-CoV-2 infected patients, affecting 108 of 175 patients (62%) that had a serum potassium of 4.0 mmol/l which is the value required for those with myocardial dysfunction.” Hypokalaemia affected 62% of patients with a K less than 3.5? Or hypokalaemia as defined by K<3.5 affected 62% of the cohort.
- “only 10 patients had a serum K >4.0 mmol/l which is the value required for those with myocardial dysfunction”. Please clarify what is meant by this phrase.
- “Principal cell” does not warrant capitalisation.
- “Although hyperkalaemia can occur for many reasons in patients with SARS-CoV-2 infection, the incidence of ventricular fibrillation has been shown to be five-fold higher in patients with hypokalaemia compared to those with hyperkalaemia ^[21]^.” This data is from the setting of acute myocardial infarction, not SARS-CoV-2, and therefore may not be applicable. Please add a qualifying statement.
- You recommend measuring the transtubular potassium gradient. Does this significantly alter management in these patients, outside of a research context?
- “It has been recommended that patients with ARDS also be kept in a relative state of volume depletion as to not worsen pulmonary interstitial oedema and thus oxygenation.” This advice has since been dropped in the context of SARS-CoV-2 – please make this clear.
- I do not think the full explanation of how loop diuretics/thiazides can cause hypokalaemia materially adds to the paper.

Is the topic of the opinion article discussed accurately in the context of the current literature?

Yes

Are arguments sufficiently supported by evidence from the published literature?

Yes

Are all factual statements correct and adequately supported by citations?

Yes

Are the conclusions drawn balanced and justified on the basis of the presented arguments?

Yes

Reviewer Expertise:

Renal tubular physiology.

We confirm that we have read this submission and believe that we have an appropriate level of expertise to confirm that it is of an acceptable scientific standard.

## References

[1] : Renal ACE2 expression in human kidney disease. *J Pathol*.2004;204(5) : 10.1002/path.1670 587-93 10.1002/path.1670 15538735

# Peer review: Reviewer response for version 2

**DOI:** 10.5256/f1000research.27922.r67707

**Published:** 2020-08-20

**Authors:** Paul Welling

**Affiliations:** 1 Johns Hopkins University, School of Medicine, Baltimore, MD, USA

I have read the revised review. It is much improved. I would accept without further revision.

Is the topic of the opinion article discussed accurately in the context of the current literature?

Partly

Are arguments sufficiently supported by evidence from the published literature?

Partly

Are all factual statements correct and adequately supported by citations?

Partly

Are the conclusions drawn balanced and justified on the basis of the presented arguments?

Partly

Reviewer Expertise:

I am an expert on molecular underpinnings of potassium balance and electrolyte disorders. I coined the "potassium switch"

I confirm that I have read this submission and believe that I have an appropriate level of expertise to confirm that it is of an acceptable scientific standard.

# Peer review: Reviewer response for version 1

**DOI:** 10.5256/f1000research.26961.r64575

**Published:** 2020-06-26

**Authors:** Paul Welling

**Affiliations:** 1 Johns Hopkins University, School of Medicine, Baltimore, MD, USA

As pointed out by this review, the evidence for hypokalemia in COVID-19 is weak, promulgated by a single unpublished article that was not corroborated by larger series studies. My chief concern about the review, is that it amplifies the myth. Furthermore, the frequent use of loop diuretics in ARDS, which cause hypokalemia, makes a causal connection between the development of hypokalemia and COVID-19 precarious.

 At the very least, the title should be changed to minimize the connection between hypokalemia and COVID-19.  

 Paragraph 1 is somewhat misleading.  Although COVID-19 does have an array of different sequela, acute respiratory distress syndrome (ARDS) seems to be the most common serve form of the disease. The first paragraph should provide an incidence of this and the other sequela. Reference to large studies from China, Europe and North American should be cited.

 Paragraph 2 The statement “SARS-CoV-2 may affect the kidneys directly and indirectly by both renal and renin-angiotensin-aldosterone system (RAS) involvement,” has no factual basis. There are a few small studies that demonstrate that SARS-CoV-2 can infect the kidney but it is presently unclear if this is a key mechanism of AKI in COVID19.

 Paragraph 3 Provide a reference to Mas receptor on type 2 alveolar epithelial cells as stated.

 Paragraph 3 last sentence. This is evidence for SARS-COV, not SARS-COV2

 Provide a reference to “hyperaldosteronism does not always cause hypokalaemia.” Although “Switch” activation has been a popular explanation for this, it has never been proven. Perhaps, the authors could cite clinical studies, demonstrating the development of severe hypokalemia in normokalemic PA patients following thiazide administration as evidence.

 The limitations of TTKG should be articulated.

Is the topic of the opinion article discussed accurately in the context of the current literature?

Partly

Are arguments sufficiently supported by evidence from the published literature?

Partly

Are all factual statements correct and adequately supported by citations?

Partly

Are the conclusions drawn balanced and justified on the basis of the presented arguments?

Partly

Reviewer Expertise:

I am an expert on molecular underpinnings of potassium balance and electrolyte disorders. I coined the "potassium switch"

I confirm that I have read this submission and believe that I have an appropriate level of expertise to state that I do not consider it to be of an acceptable scientific standard, for reasons outlined above.
